# Serum Npas-4 and Nptx-2 Levels in Alzheimer’s Disease: Potential Biomarkers of Synaptic Dysfunction in a Cross-Sectional Study

**DOI:** 10.3390/biom15060795

**Published:** 2025-05-30

**Authors:** Alev Lazoglu Ozkaya, Nilifer Gürbüzer, Tolga Mercantepe, Filiz Mercantepe

**Affiliations:** 1 Department of Biochemistry, Erzurum City Hospital, Erzurum 25240, Türkiye; alev.lazogluozkaya@saglik.gov.tr; 2 Department of Psychiatry, Erzurum Faculty of Medicine, University of Health Sciences, Erzurum 25240, Türkiye; nilifer.gurbuzer@sbu.edu.tr; 3 Department of Histology, Faculty of Medicine, Recep Tayyip Erdogan University, Rize 53100, Türkiye; 4 Department of Endocrinology and Metabolism, Faculty of Medicine, Recep Tayyip Erdogan University, Rize 53100, Türkiye; filiz.mercantepe@saglik.gov.tr

**Keywords:** Alzheimer’s disease, NPAS-4, NPTX-2, synaptic plasticity, biomarker, lipid metabolism

## Abstract

**Background:** Alzheimer’s disease (AD) is a progressive neurodegenerative disorder characterized by cognitive decline, synaptic dysfunction, and neuronal loss. Identifying reliable biomarkers for early diagnosis and disease monitoring remains a critical need. **Objective:** This study aimed to investigate the serum levels of NPAS-4 (Neuronal PAS Domain Protein 4) and NPTX-2 (Neuronal Pentraxin 2) in patients with Alzheimer’s disease, exploring their potential roles in disease pathophysiology and their relationship with lipid parameters. **Methods:** This was a cross-sectional study that included 63 patients diagnosed with Alzheimer’s disease and 56 age- and sex-matched healthy controls. Venous blood samples were collected from all participants. NPAS-4 and NPTX-2 levels were measured using the ELISA method, while lipid parameters were analyzed via spectrophotometric techniques. Cognitive assessment was performed using the Standardized Mini-Mental Test (SMMT). Comparative analyses between groups, correlation studies, logistic regression, and ROC analyses were conducted. **Results:** Serum NPAS-4 and NPTX-2 levels were significantly lower in Alzheimer’s patients compared to healthy controls (*p* < 0.001 and *p* = 0.001, respectively). Additionally, total cholesterol and LDL levels were lower in the patient group. Logistic regression analysis identified NPAS-4 as an independent risk predictor for Alzheimer’s disease (OR = 0.313, *p* < 0.001). ROC analyses demonstrated that both biomarkers had significant diagnostic discrimination power. However, no significant correlation was found between NPAS-4 and NPTX-2 levels and SMMT scores or lipid parameters. **Conclusions:** The decreased levels of NPAS-4 and NPTX-2 in Alzheimer’s patients may reflect biochemical manifestations of impaired synaptic plasticity. These findings suggest that NPAS-4 and NPTX-2 may serve as potential early biomarkers in the diagnosis and monitoring of Alzheimer’s disease.

## 1. Introduction

Alzheimer’s disease (AD) is a neurodegenerative disorder and is considered the most common cause of progressive cognitive decline and dementia [1]. Clinical findings can progress from mild forgetfulness and distractibility in the early stages to complete dependence, dysphagia, and motor losses in advanced stages [2]. The fundamental pathophysiological processes of the disease include the accumulation of beta-amyloid (Aβ) in the brain, hyperphosphorylation of tau proteins, synaptic plasticity dysfunction, neuroinflammation, and oxidative stress, along with widespread neuronal loss, which is also a hallmark pathological feature of Alzheimer’s disease [3,4]

The prevalence of Alzheimer’s disease increases with age, reaching 25–50% in individuals over 85 years old [5]. Approximately 50 million people worldwide are affected by dementia, with an estimated 60–70% diagnosed with Alzheimer’s disease [6]. While the cause of the disease is generally multifactorial, a small portion is associated with early-onset forms related to mutations in the *APP*, *PSEN1*, and *PSEN2* genes [7]. The chronic and progressive nature of the disease poses a significant health burden at both individual and societal levels. In this context, the importance of biomarkers that can be measured in blood and cerebrospinal fluid for early diagnosis and monitoring of the disease is increasingly recognized [8].

Synaptic dysfunction is one of the main causes of cognitive decline in Alzheimer’s disease [9]. NPTX-2 is a protein released from glutamatergic neurons that plays a role in synaptic plasticity and intercellular signal transmission [10]. This protein contributes to maintaining the excitatory–inhibitory balance through its interaction with GABAergic PV^+^ interneurons [11,12,13]. A deficiency in NPTX-2 can disrupt this balance, leading to impairments in learning and memory functions. Additionally, NPTX-2 has been reported to play a role in the regulation of neuroinflammation [14]. Changes in NPTX-2 levels have also been demonstrated in neuropsychiatric disorders such as epilepsy and schizophrenia. Interestingly, it has been suggested that microglial complement activation, which is effective in synaptic loss in diseases like Alzheimer’s and frontotemporal dementia, may be associated with NPTX-2 [15].

NPAS-4, on the other hand, is a transcription factor that is expressed in glutamatergic neurons in response to synaptic activity and regulates the formation of inhibitory synapses [16]. It shows the highest expression in the frontal cortex and hippocampus. NPAS-4 helps maintain the excitatory–inhibitory balance by supporting GABAergic synapses [17,18]. It also increases the expression of neurotrophic factors such as BDNF and provides neuronal protection against oxidative stress [19]. A deficiency in NPAS-4 is associated with both synaptic plasticity dysfunction and weakened neuronal defense mechanisms [20]. Therefore, the role of NPAS-4 in understanding synaptic losses and neuroinflammation in Alzheimer’s disease is becoming increasingly important.

It has long been known that cholesterol metabolism in the brain is impaired in Alzheimer’s disease. Cholesterol is a critical molecule for synaptic membrane structure, vesicle fusion, neurotransmitter release, and receptor functions [21]. The development of new biomarkers that can be studied in serum, alongside traditional biomarkers measured in cerebrospinal fluid such as Aβ, p-Tau, and t-Tau, offers more accessible and non-invasive diagnostic possibilities.

This study hypothesized that the levels of NPAS-4 and NPTX-2 would be lower in patients with Alzheimer’s disease compared to healthy individuals, and that these biomarkers would be associated with disease-related pathophysiological processes.

In this context, investigating serum levels of NPAS-4 and NPTX-2 may provide valuable insights for the early diagnosis of Alzheimer’s disease and monitoring disease progression. Additionally, determining the relationship of these proteins with clinical data can contribute to a better understanding of the biological foundations of the disease. Therefore, the possibility that NPAS-4 and NPTX-2 may serve as diagnostic and therapeutic biomarkers in Alzheimer’s disease warrants further investigation.

## 2. Materials and Methods

Patients who presented to the Psychiatry outpatient clinic of Erzurum City Hospital between 12 December 2024 and 30 March 2025, and were diagnosed with probable Alzheimer’s disease according to the DSM-5 and the diagnostic criteria established by the National Institute on Aging–Alzheimer’s Association (NIA-AA) working groups, based on their history, psychiatric and neurological examination, were included in the study [2,22,23]. Secondary causes of dementia were excluded through blood samples and cranial magnetic resonance imaging. The control group consisted of healthy volunteers who were the same age and gender as the patient group, had cognitive test results that did not meet dementia criteria, had no comorbidities or regular medication use, and were examined in our outpatient clinic for other reasons during the same period. Written informed consent was obtained from all participants and/or their guardians.

The research protocol of the study was approved by the Scientific Research Ethics Committee of Erzurum Medical Faculty, Health Sciences University (Erzurum, Turkey) with decision number BAEK 2024/12-233 and was conducted in accordance with the Helsinki Declaration.

### 2.1. Research Sample

In our study, we evaluated the participants’ cross-sectional clinical data, lipid profiles, and fasting serum levels of NPAS-4 and NPTX-2. The study included 63 patients with probable Alzheimer’s disease who had not previously received anti-dementia treatment. All patients underwent the Standardized Mini-Mental Test (SMMT) to objectively assess cognitive status. A score below 24 points on the SMMT was interpreted as indicative of cognitive impairment consistent with dementia.

The control group consisted of 56 healthy participants who were the same age and gender as the patient group, examined in our outpatient clinic for other reasons, and who met the inclusion and exclusion criteria, with blood samples taken. All members of the control group were healthy individuals with no comorbidities or regular medication use, who underwent the SMMT and scored 24 points or higher.

### 2.2. Procedure

The age range of participants was 65–85 years. There were no gender restrictions among participants. The exclusion criteria for both patients and healthy controls included obesity (Body Mass Index (BMI) > 30 kg/m^2^), a history of major psychiatric disorders that began before the onset of neurocognitive disorders (such as major depression, bipolar disorder, and schizophrenia), the presence of comorbid neurological and systemic diseases (such as cerebrovascular events, Parkinson’s disease, epilepsy, heart disease, lung or kidney failure, hematological diseases, malignancy, connective tissue diseases, acute/chronic inflammatory or autoimmune diseases), a history of infection in the last two weeks, and a history of immunosuppressive, anticoagulant, or anti-inflammatory drug use.

Sociodemographic Data Form. This is a form developed by researchers to record characteristics such as age, gender, height, weight, and Body Mass Index (BMI) of both the patient and control groups.

Standardized Mini Mental Test (SMMT). Developed by Folstein et al., this is a short and useful screening test administered by the interviewer to assess general cognitive functions [24]. The scale consists of 11 items under five main headings: orientation, registration memory, recall, attention and calculation, and language. It is scored between 0 and 30. A validity and reliability study of the scale in Turkish has been conducted [25]. In the Turkish sample, a cut-off score of 24 points was determined for mild-stage dementia. This cut-off was found to have very high sensitivity (0.91) and specificity (0.95) for the diagnosis of mild dementia [25]. SMMT scores were classified to assess the level of cognitive impairment. In this context, scores below 24 were considered indicative of cognitive impairment, 23–20 as mild, 19–10 as moderate, and 0–9 as severe.

### 2.3. Biochemical Analysis

Participants were instructed to rest in a seated position between 08:00 and 10:00 AM, after which venous blood samples were collected from the antecubital area for the analysis of total cholesterol, HDL, LDL, triglycerides, NPAS-4, and NPTX-2 parameters. After allowing the samples to clot at room temperature for 30 min, serum was separated by centrifugation at 3000 rpm for 10 min. Total cholesterol, HDL, LDL, and triglyceride levels were determined using the Siemens Atellica^®^ (Siemens Healthineers, Erlangen, Germany) clinical chemistry analyzer by spectrophotometric methods. Serum samples were aliquoted and stored at −80 °C until the day of the Elisa study. After ensuring suitable conditions for analysis, each sample was analyzed in duplicate to complete the study. Elisa studies were conducted at the Medical Biochemistry Laboratory of Erzurum City Hospital. Serum samples for NPAS-4 and NPTX-2 measurements were analyzed using BT Lab^®^ Elisa kits (BT Lab, Human Neuronal PAS domain-containing protein 4, Cat Log No: E6793Hu; Human Neuronal Pentraxin 2, Cat Log No: E4709Hu, Jiaxing Korain Biotech, Jiaxing, China) according to the manufacturer’s recommended standard protocol, using the Rel Assay^®^ automatic Elisa reader (Biobase Biodusty Co., Ltd., Jinan, Shandong, China). The measurement range for the kits was 0.05–30 ng/mL for NPAS-4 and 0.05–20 ng/mL for NPTX-2.

### 2.4. Statistical Analysis

All statistical analyses were performed using IBM SPSS Statistics version 22.0 (IBM Corp., Armonk, NY, USA). The normality of continuous variables was assessed by evaluating skewness and kurtosis values, all of which fell within the acceptable range of −2 to +2, indicating normal distribution assumptions were met [26]. Continuous variables were expressed as mean ± standard deviation, and categorical variables as numbers and percentages. Comparisons between categorical variables were performed using the Chi-square test. For comparisons between two independent groups, the independent samples *t*-test was applied due to the normal distribution of data. Effect sizes between groups were calculated using Cohen’s d statistic. For comparisons involving more than two independent groups of continuous variables, One-Way ANOVA was applied, as the assumption of normal distribution was met. Following the ANOVA, post hoc analyses were conducted using the Bonferroni test when the assumption of homogeneity of variances was satisfied, and Tamhane’s T2 test when this assumption was violated. Relationships between continuous variables were evaluated using Pearson correlation analysis. In multivariate analysis, logistic regression was performed to identify predictive risk factors between groups, based on the potential risk variables identified in univariate analysis. Receiver Operating Characteristic (ROC) curve analysis was conducted to assess the diagnostic performance and determine optimal cut-off points for continuous variables. A *p*-value of <0.05 was considered statistically significant.

Post hoc power analysis was performed using G Power 3.1 software. Based on the observed means and standard deviations in our study, the calculated effect size for NPAS-4 was 0.81, and for NPTX-2, it was 0.62. For a significance level of 0.05 and group sizes of 63 and 56, the statistical power was 99.9% and 97.5%, respectively.

## 3. Results

A total of 119 participants were included in this study, consisting of 63 patients and 56 healthy controls. Among the patients, 43 (68.3%) were female, while 36 (64.3%) of the controls were female. No significant differences were observed between the patient and control groups in terms of age, gender, and BMI. The mean SMMT score in the patient group was 15.22 ± 4.73, indicating cognitive impairment. Serum levels of LDL (*p* = 0.013), total cholesterol (*p* < 0.001), NPAS-4 (*p* < 0.001), and NPTX-2 (*p* = 0.001) were significantly lower in the patient group compared to healthy controls (Table 1).

Participants with SMMT scores between 23 and 20 were classified as having mild cognitive impairment, those scoring between 19 and 10 as having moderate cognitive impairment, and those scoring between 0 and 9 as having severe cognitive impairment. In the study, 15 individuals had mild cognitive impairment, 38 had moderate cognitive impairment, and 10 had severe cognitive impairment. In the comparison of the three groups, only age showed a significant difference (*p* = 0.005, F = 5.698). The age of patients with mild cognitive impairment was significantly lower than those with moderate and severe cognitive impairment. The mean age of patients with mild cognitive impairment was 74.20 ± 3.51 years, while it was 78.24 ± 5.74 years for those with moderate cognitive impairment and 80.90 ± 4.41 years for those with severe cognitive impairment (Table 2).

In the patient group, a moderate and statistically significant negative correlation was observed between age and the severity of cognitive impairment based on SMMT scores (r = −0.527, *p* < 0.001). However, no significant correlations were found between clinical and biochemical parameters and the levels of NPAS-4 and NPTX-2 (Table 3).

Risk factors for probable Alzheimer’s disease in participants aged 65–85 were evaluated using logistic regression analysis (Backward Conditional Model). In the model, Alzheimer’s disease was the dependent variable, while age, gender, BMI, NPAS-4, and NPTX-2 levels were used as independent variables. In this assessment, NPAS-4 (OR: 0.31, 95% CI [0.175, 0.559]) levels were identified as a risk factor for probable Alzheimer’s disease (Table 4).

**Figure 1:**
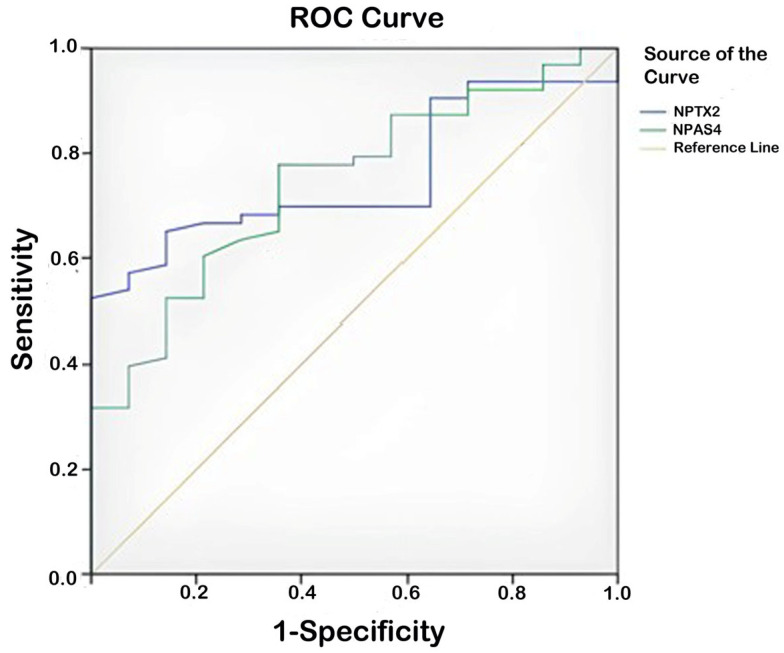
ROC curve analysis for NPAS-4 and NPTX-2 (patient group–control group); NPAS-4 (cut-off value 2.41, sensitivity 77.8%, specificity 64.3%); NPTX-2 (cut-off value 2.34, sensitivity 68.3%, specificity 64.3%).

ROC analysis was conducted to determine the role of NPAS-4 and NPTX-2 in predicting probable Alzheimer’s disease. The analysis results showed that NPAS-4 (AUC ± SE; 95% CI; 0.744 ± 0.045; 0.656–0.831) and NPTX-2 (AUC ± SE; 95% CI; 0.755 ± 0.046; 0.665–0.845) could be used to predict the disease. Low levels of NPAS-4 and NPTX-2 increased the likelihood of predicting probable Alzheimer’s disease. The cut-off value for NPAS-4 in predicting the disease was found to be 2.41 (sensitivity 77.8%, specificity 64.3%), and the cut-off value for NPTX-2 was 2.34 (sensitivity 68.3%, specificity 64.3%) (Table 5, Figure 1).

## 4. Discussion

Alzheimer’s disease is a progressive neurological disorder characterized by cognitive function loss, synaptic dysfunction, and widespread neurodegeneration, for which there is currently no definitive treatment. Various mechanisms are involved in its pathophysiology, including the accumulation of amyloid beta (Aβ), hyperphosphorylation of tau proteins, neuroinflammation associated with microglial activation, and excitatory–inhibitory synaptic imbalance [4]. These processes lead to a disruption of synaptic integrity and communication breakdowns in neuronal networks, resulting in significant cognitive impairment. Maintaining synaptic plasticity and neuronal homeostasis is crucial for sustaining higher cognitive functions such as learning and memory [17].

NPAS-4 is a transcription factor whose expression increases in response to neuronal activity and regulates the formation of inhibitory synapses [16]. In response to excitatory synaptic activity, NPAS-4 expression rises within neurons, promoting the formation of inhibitory synapses through GABAergic (particularly PV^+^) interneurons [18]. Thus, it contributes to maintaining the excitatory–inhibitory balance. On the other hand, NPTX-2 is a synaptic organization protein released by glutamatergic neurons that ensures the stability of excitatory synapses and the regulation of connections with PV^+^ interneurons [1]. Both molecules play significant roles in synaptic communication as regulators and stabilizers, essential for the healthy functioning of neuronal networks and the sustainability of cognitive functions.

In our study, serum NPAS-4 levels in Alzheimer’s patients were found to be significantly lower compared to healthy controls. The decrease in NPAS-4 levels may be associated with synaptic plasticity impairment, a characteristic feature of the disease, and the disruption of the excitatory–inhibitory balance. The loss of synaptic stability and the reduction in inhibitory synapse formation can lead to excessive excitatory signals and thus toxicity in cortical and hippocampal circuits responsible for learning and memory [18,19]. Various studies in the literature have shown that the activity of the GABAergic system is reduced in Alzheimer’s disease and that the functions of inhibitory interneurons are impaired [27]. Considering these findings, the decline in NPAS-4 levels is critically important regarding both the inadequacy in suppressing excitatory signals and the disruption of synaptic homeostasis.

Additionally, NPAS-4 is thought to be involved in neuroinflammation and cell death processes associated with Alzheimer’s disease. It has been shown that NPAS-4 deficiency increases the activity of microglia and astrocytes, raising levels of pro-inflammatory cytokines such as IL-6 and TNF-α. Furthermore, the loss of NPAS-4 may trigger a transition from apoptotic processes to necrotic cell death by altering cellular death mechanisms [28]. In this context, NPAS-4 can be regarded not only as a factor limiting synaptic plasticity but also as a neuroprotective element that restricts neuroinflammation.

NPAS-4 has been shown to function in various neurological and psychiatric disorders beyond Alzheimer’s disease. In mouse models where the NPAS-4 gene is completely deleted, various behavioral disorders such as hyperactivity, reduced social interaction, loss of cognitive flexibility, and impaired pre-pulse inhibition have been reported [29]. Additionally, it has been reported that NPAS-4 deficiency is associated with reduced lifespan and increased neurodegeneration [30]. In depression models, a decrease in NPAS-4 expression, particularly in the hippocampal region, has been demonstrated, and this reduction has been associated with mood regulation [31]. In NPAS-4 heterozygous mice exposed to stress during adolescence, the decrease in this protein’s expression led to cognitive dysfunctions in adulthood [32]. Furthermore, in mice lacking NPAS-4 after ischemic stroke, increased anxiety levels and impairments in social behavior have been observed [33]. In human studies, significantly lower levels of NPAS-4 have been found in peripheral blood mononuclear cells of patients with post-stroke depression [34]. It has also been shown that individuals with high hippocampal NPAS-4 levels exhibit more balanced physiological responses to stress [35]. All these data indicate that NPAS-4 is closely related not only to synaptic organization and cognitive functions but also to the regulation of emotional responses and mechanisms for coping with stress. The decrease in NPAS-4 levels observed in Alzheimer’s patients in our study may be associated not only with cognitive impairments but also with potential emotional and behavioral symptoms.

Moreover, the logistic regression analyses obtained in our study revealed that NPAS-4 levels are an independent and strong risk predictor for Alzheimer’s disease. According to the Backward Conditional Model, as NPAS-4 levels decrease, the likelihood of Alzheimer’s significantly increases, highlighting NPAS-4 as a significant protective factor. Additionally, the values obtained from our ROC analysis suggest that NPAS-4 may possess clinically significant discriminatory power in the diagnosis of Alzheimer’s. These results support the notion that NPAS-4 could be a biomarker that should be considered not only in pathophysiological processes but also at the diagnostic level.

When examining NPTX-2 levels, our study found that serum NPTX-2 levels in Alzheimer’s patients were significantly lower compared to healthy controls. This decrease may serve as a biochemical indicator of the synaptic loss and weakening of glutamatergic networks that begin early in Alzheimer’s disease. In the literature, it has been shown that NPTX-2 levels in cerebrospinal fluid decrease significantly across all stages of Alzheimer’s, and this reduction is more pronounced than that of other synaptic proteins [36]. Furthermore, it has been reported that NPTX-2 levels are positively correlated with hippocampal volume, and this relationship may be directly linked to cognitive performance [37]. The deficiency of NPTX-2 may particularly weaken synaptic connections with parvalbumin-positive interneurons, leading to disruptions in cortical rhythms. This situation may adversely affect learning and memory as well as information processing.

Recent animal studies provide new evidence supporting the potential role of NPTX-2 in Alzheimer’s disease. In a rat model, baicalin—a natural compound—was shown to increase NPTX-2 levels, accompanied by improvements in learning and memory performance. These findings suggest that NPTX-2 may be a promising target not only for diagnosis but also for therapeutic intervention. In our study, significantly lower NPTX-2 levels in Alzheimer’s patients further support its involvement in the disease process and highlight its potential as a candidate for future treatment strategies [38]

The role of NPTX family proteins in regulating synaptic network dynamics is not limited to Alzheimer’s disease. In disorders characterized by excitatory–inhibitory imbalance, such as schizophrenia, decreased levels of NPTX-2 have also been demonstrated. Analyses of prefrontal cortex tissues from schizophrenia patients have reported significant reductions in the expression of genes associated with the GABAergic system, such as NPTX-2 and parvalbumin [39,40]. These findings suggest that NPTX-2 may be regarded as a universal regulator that supports the integrity of inhibitory circuits.

Although our study found that NPTX-2 levels were significantly lower in Alzheimer’s patients compared to healthy controls, it did not show statistical significance as an independent risk predictor in the logistic regression analysis. This suggests that the potential of NPTX-2 to strongly predict the presence of the disease alone may be limited. However, the values calculated for NPTX-2 in the ROC analysis indicate that there is a statistically significant, albeit limited, discriminatory power for diagnosis. These findings suggest that NPTX-2 may be considered as a supportive biomarker in the diagnosis of Alzheimer’s disease, but further studies are needed for its standalone use.

Additionally, our study found that serum total cholesterol and LDL levels in Alzheimer’s patients were significantly lower than those in healthy individuals. However, no significant differences were found in HDL and triglyceride levels between the groups. There was also no significant correlation between SMMT scores and lipid parameters. Considering the role of cholesterol in synaptic membrane stability, neuronal signal transmission, and Aβ metabolism, low cholesterol levels may contribute to impaired synaptic functions in Alzheimer’s patients [41,42]. Some studies suggest that low cholesterol levels are associated with cognitive decline [41], while others argue that high cholesterol levels may be an independent risk factor for Alzheimer’s disease, with the *ApoE ε4* allele potentially mediating this effect through cholesterol [43,44]. These conflicting results may be explained by factors such as population differences, genetic predisposition, dietary habits, and the diagnostic criteria used.

Our study is one of the limited numbers of research efforts that evaluate NPAS-4 and NPTX-2 levels in Alzheimer’s patients together. It is also significant as one of the pioneering studies examining the relationship of these biomarkers with lipid parameters. The appropriate matching of groups in terms of age and gender has increased the reliability of the statistical analyses. Additionally, the serum protein levels obtained through the ELISA method support the accuracy of biochemical data. While this study is one of the preliminary works assessing NPAS-4 and NPTX-2 levels in Alzheimer’s patients, it has some limitations. Although strict inclusion and exclusion criteria were applied, the fact that the data were collected based on hospital records raises the possibility that undiagnosed comorbidities or unrecorded treatments may have been overlooked. Furthermore, although individuals not receiving anti-dementia treatment and those with Alzheimer’s diagnosis were included in the study, it is not fully known how long the symptoms of the disease have been present in these individuals, which could be a limiting factor in evaluating the relationship between biomarker levels and the stage of the disease. Additionally, variables that could affect biomarker levels, such as participants’ nutritional status, metabolic balance, and micronutrient deficiencies, could not be systematically assessed. This study only compared Alzheimer’s patients with healthy individuals; therefore, a comparison of NPAS-4 and NPTX-2 levels regarding disease specificity could not be made with other types of dementia, such as vascular dementia, Lewy body dementia, and frontotemporal dementia. Moreover, due to the cross-sectional nature of our study, the dynamic effects of NPAS-4 and NPTX-2 levels at the onset or during the progression of the disease could not be evaluated. The sample size does not allow for the analysis of genetic factors such as *ApoE* genotype or additional variables like CSF biomarkers. For these reasons, the generalizability of the findings should be interpreted with caution, and there is a need for larger, multicenter studies in the future.

## 5. Conclusions

In this study, we evaluated serum NPAS-4 and NPTX-2 levels in Alzheimer’s patients to investigate the possible roles of these molecules in disease-related synaptic changes. Our findings indicate that both proteins are significantly lower in Alzheimer’s patients compared to healthy controls, suggesting that this decrease may be associated with synaptic plasticity impairment. This situation implies that NPAS-4 and NPTX-2 could be early biochemical markers of the neurodegenerative process.

When evaluated in terms of serum lipid profile, total cholesterol and LDL levels were observed to be low in the Alzheimer’s group. While this finding suggests a potential change in lipid metabolism in Alzheimer’s patients, no direct correlation was found between NPAS-4 and NPTX-2 levels and lipid parameters. Similarly, the lack of a significant relationship between these biomarkers and SMMT scores indicates that changes in these molecules may occur in the early stages of the disease, even before clinical symptoms emerge.

Our findings support the hypothesis that NPAS-4 and NPTX-2 are involved in the pathophysiological process of Alzheimer’s disease and may serve as potential biomarkers for early diagnosis and monitoring of disease progression. However, to better understand their clinical utility and diagnostic value, future longitudinal and multicenter studies with larger sample sizes are needed.

## Figures and Tables

**Table 1:** Comparison of clinical and blood parameters between patient and control groups.

	Patient Group *n* = 63	Control Group *n* = 56	t	Effect Size	*p*
	Mean ± SD	Mean ± SD			
Age (year)	77.70 ± 5.48	76.13 ± 5.57	−1.552		0.123
BMI (kg/m^2^)	25.27 ± 3.22	25.35 ± 2.71	0.140		0.889
SMMT	15.22 ± 4.73	25.75 ± 1.32	16.925	3.03	<0.001
Triglyceride (mg/dL)	135.21 ± 56.60	146.71 ± 49.38	1.175		0.242
HDL Cholesterol (mg/dL)	42.55 ± 10.87	45.05 ± 8.05	1.436		0.154
LDL Cholesterol (mg/dL)	121.37 ± 36.19	137.50 ± 32.81	2.536	0.47	0.013
Total Cholesterol (mg/dL)	173.24 ± 41.01	203.50 ± 31.91	4.452	0.82	<0.001
NPAS-4 (ng/dL)	2.12 ± 0.82	2.81 ± 0.89	4.387	0.81	<0.001
NPTX-2 (ng/dL)	2.35 ± 1.24	3.08 ± 1.11	3.337	0.62	0.001

Note: *p* < 0.05: statistical significance level in comparison of groups; BMI: Body Mass Index; effect size = Cohen’s d (0.2—small; 0.5—medium; and 0.8—large effect size); HDL: high-density lipoprotein; LDL: low-density lipoprotein; Mean ± SD: mean ± standard deviation; NPAS-4: Neuronal PAS Domain Protein 4; NPTX-2: Neuronal Pentraxin 2; SMMT: Standardized Mini-Mental Test; *n*: number of participants; t: independent sample *t*-test.

**Table 2:** Comparison of clinical and blood parameters of patients with mild, moderate, and severe cognitive impairment.

	Mild Cognitive Impairment *n* = 15	Moderate Cognitive Impairment *n* = 38	Severe Cognitive Impairment *n* = 10	F	*p*
	**Mean ± SD**	**Mean ± SD**	**Mean ± SD**		
Age (year)	74.20 ± 3.51 ^a^	78.24 ± 5.74 ^b^	80.90 ± 4.41 ^bc^	5.698	0.005
BMI (kg/m^2^)	24.06 ± 3.18	25.83 ± 3.01	24.95 ± 3.83	1.716	0.189
Triglyceride (mg/dL)	141.80 ± 59.28	135.21 ± 57.67	125.30 ± 52.42	0.249	0.781
HDL Cholesterol (mg/dL)	45.02 ± 12.53	42.75 ± 10.09	38.13 ± 10.89	1.231	0.299
LDL Cholesterol (mg/dL)	124.47 ± 31.32	123.29 ± 39.43	109.40 ± 30.29	0.648	0.527
Total Cholesterol (mg/dL)	174.13 ± 46.20	177.95 ± 39.19	154.00 ± 38.02	1.371	0.262
NPAS-4 (ng/dL)	2.06 ± 0.85	2.07 ± 0.73	2.38 ± 1.12	0.597	0.553
NPTX-2 (ng/dL)	2.37 ± 1.45	2.24 ± 0.94	2.75 ± 1.85	0.686	0.508

Note: *p* < 0.05: statistical significance level in comparison of groups; ^a, b, c^: significance between three groups; BMI: Body Mass Index; F: One-Way ANOVA test value; HDL: high-density lipoprotein; LDL: low-density lipoprotein; Mean ± SD: mean ± standard deviation; *n*: number of participants; NPAS-4: Neuronal PAS Domain Protein 4; NPTX-2: Neuronal Pentraxin 2.

**Table 3:** Correlation of clinical and blood parameters in the patient group.

		1	2	3	4	5	6	7	8	9
Age	r	1								
	*p*									
BMI	r	−0.082	1							
	*p*	0.520								
SMMT	r	−0.527	−0.147	1						
	*p*	0.000	0.250							
TG	r	−0.105	−0.140	0.111	1					
	*p*	0.413	0.273	0.388						
HDL	r	−0.096	0.029	0.211	−0.141	1				
	*p*	0.453	0.820	0.097	0.269					
LDL	r	0.090	0.045	0.082	0.486	0.167	1			
	*p*	0.483	0.728	0.525	0.000	0.191				
TC	r	0.009	−0.010	0.144	0.442	0.479	0.787	1		
	*p*	0.946	0.940	0.262	0.000	0.000	0.000			
NPAS-4	r	−0.042	−0.219	−0.055	0.210	−0.096	0.064	−0.008	1	
	*p*	0.747	0.085	0.668	0.098	0.455	0.617	0.951		
NPTX-2	r	−0.036	−0.131	−0.060	0.205	0.015	0.033	0.109	0.783	1
	*p*	0.782	0.306	0.643	0.107	0.907	0.800	0.395	0.000	

Note: *p* < 0.05: statistical significance level; BMI: Body Mass Index; HDL: high-density lipoprotein; LDL: low-density lipoprotein; TC: total cholesterol; NPAS-4: Neuronal PAS Domain Protein 4; NPTX-2: Neuronal Pentraxin 2; SMMT: Standardized Mini-Mental Test.

**Table 4:** Risk factors for Alzheimer’s disease—logistic regression Backward Conditional Model.

Risk Factors		B	S.E.	*p*	Exp(B)	95% C.I. for EXP(B)	
						Lower	Upper
Step 1 ^a^	Age	0.054	0.037	0.146	1.056	0.981	1.136
	Gender(1)	−0.242	0.449	0.590	0.785	0.325	1.894
	BMI	−0.128	0.076	0.090	0.880	0.759	1.020
	NPAS-4	−1.150	0.380	0.003	0.317	0.150	0.668
	NPTX-2	−0.022	0.243	0.929	0.979	0.608	1.575
	Constant	2.090	3.55	0.556	8.086		
Step 4 ^a^	BMI	−0.121	0.073	0.096	0.886	0.768	1.022
	NPAS-4	−1.163	0.297	0.000	0.313	0.175	0.559
	Constant	6.002	2.224	0.007	404.286		

Note: *p* < 0.05: statistical significance level; BMI: Body Mass Index; NPAS-4: Neuronal PAS Domain Protein 4; NPTX-2: Neuronal Pentraxin 2; (1): Female; a: Variable(s) entered on step 1 age, BMI, gender, Nptx-2, Npas-4

**Table 5:** Area under the curve.

Test Result Variable(s)		Area	Std. Error ^a^	Asymptotic Sig. ^b^	Asymptotic 95% Confidence Interval	
					Lower Bound	Upper Bound
Patient group–control group	NPAS-4	0.744	0.045	0.000	0.656	0.831
	NPTX-2	0.755	0.046	0.000	0.665	0.845

Note: *p* < 0.05; statistical significance level; ^a^ under the nonparametric assumption; ^b^ null hypothesis: true area = 0.5.

## Data Availability

All data generated or analyzed during this study are included in this article. The data will be available upon reasonable request (contact tolga.mercantepe@erdogan.edu.tr).
